# Microbial host selection affects intracellular localization and activity of alcohol-O-acetyltransferase

**DOI:** 10.1186/s12934-015-0221-9

**Published:** 2015-03-17

**Authors:** Jie Zhu, Jyun-Liang Lin, Leidy Palomec, Ian Wheeldon

**Affiliations:** Department of Biochemistry, University of California, Riverside, USA 92521; Department of Chemical and Environmental Engineering, University of California, Riverside, USA 92521

**Keywords:** AATase, Enzymes, Esters, Ethyl acetate, Protein localization

## Abstract

**Background:**

A key pathway for ester biosynthesis in yeast is the condensation of an alcohol with acetyl-CoA by alcohol-O-acetyltransferase (AATase). This pathway is also prevalent in fruit, producing short and medium chain volatile esters during ripening. In this work, a series of six AATases from *Saccharomyces* and non-*Saccharomyces* yeasts as well as tomato fruit were evaluated with respect to their activity, intracellular localization, and expression in *Saccharomyces cerevisiae* and *Escherichia coli* cell hosts. The series of AATases includes Atf1 and Atf2 from *S. cerevisiae*, as well as AATases from *S. pastorianus*, *Kluyveromyces lactis*, *Pichia anomala*, and *Solanum lycopersicum* (tomato).

**Results:**

When expressed in *S. cerevisiae,* Atf1, Atf2, and an AATase from *S. pastorianus* localized to lipid droplets, while AATases from non-*Saccharomyces* yeasts and tomato fruit did not localize to intracellular membranes and were localized to the cytoplasm. All AATases studied here formed intracellular aggregates when expressed in *E. coli*, and western blot analysis revealed that expression levels in *E. coli* were upwards of 100-fold higher than in *S. cerevisiae*. Fermentation and whole cell lysate activity assays of the two most active AATases, Atf1 from *S. cerevisiae* and an AATase from tomato fruit, demonstrated that the aggregates were enzymatically active, but with highly reduced specific activity in comparison to activity in *S. cerevisiae*. Activity was partially recovered at lower expression levels, coinciding with smaller intracellular aggregates. *In vivo* and *in vitro* activity assays from heterologously expressed Atf1 from *S. cerevisiae*, which localizes to lipid droplets under homologous expression, demonstrates that its activity is not membrane dependent.

**Conclusions:**

The results of these studies provide important information on the biochemistry of AATases under homologous and heterologous expression with two common microbial hosts for biochemical processes, *S. cerevisiae* and *E. coli*. All studied AATases formed aggregates with low enzymatic activity when expressed in *E. coli* and any membrane localization observed in *S. cerevisiae* was lost in *E. coli*. In addition, AATases that were found to localize to lipid droplet membranes in *S. cerevisiae* were found to not be membrane dependent with respect to activity.

**Electronic supplementary material:**

The online version of this article (doi:10.1186/s12934-015-0221-9) contains supplementary material, which is available to authorized users.

## Background

**Figure 1:**
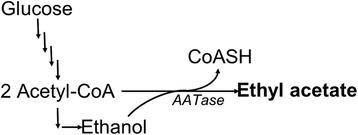
**Schematic of AATase pathway for ester biosynthesis.**

During yeast fermentation and fruit ripening short chain linear and branched alcohols are converted to their corresponding acetate esters by alcohol-O-acetyltransferase (AATase; EC 2.3.1.84; Figure 1). These volatile esters produce sweet and fruity fragrances: phenyl ethyl acetate smells of flowers, isoamyl acetate (isopentyl acetate) smells of bananas, and ethyl acetate smells of sweet pears. In plants, these and other esters function as attractors to pollinating species and as a defense mechanism, attracting predators to animals feeding on their leaves and fruit [1,2]. The function of microbial ester biosynthesis is not as well understood. AATase activity in *Saccharomcyes cerevisiae* is repressed by oxygen and unsaturated fatty acids [3-5] and it has been suggested that this activity functions as a means of CoA recycling with the co-production of organic acids [6,7], possibly as a response to stress conditions [8].

While there is uncertainty in the biological function of AATase activity in yeast, there are clear roles in metabolic engineering and industrial fermentations. The ester products have value as natural food additives, as aroma and flavor compounds in fermented beverages, and as industrial solvents [7,9]. The effects of AATase activity on aroma and flavor profiles in wine, beer, and sake fermentations are well understood [5,10-12]. The most well-studied AATases, Atf1 and Atf2 from *S. cerevisiae*, have been used to engineer whole cell *E. coli* catalysts for the conversion of ethanol and isoamyl alcohol to ethyl and isoamyl acetate [13-15] and for the biosynthesis of C4 to C11 volatile esters in *E. coli* [16]. An AATase from strawberry fruit (*Fragaria* species) has also been heterologously expressed in *E. coli* for the biosynthesis of butyl acetate and a range of butyrate esters [15,17]. Titers from these processes range from 0.04 – 0.23 g/L [13,15,17] to upwards of 17.5 g/L [16] and are, in part, limited by low AATase activity. In addition, the hydrophobic nature of these enzymes and varied intracellular localization of orthologs in their native hosts present complicating factors for heterologous expression in engineered hosts [8,18,19].

We have previously shown that Atf1 and −2 from *S. cerevisiae* localize to lipid droplets (LDs) via N- and C-terminal amphipathic helices [19]. The AATase ortholog from *S. pastorianus* also localizes to LDs by a similar mechanism, while AATases from non-*Saccharomyces* yeasts and fruit species, including *Cucumis melo* (melon), and *Solanum lycopersicum* (tomato) that do not have the conserved terminal helices from *S. cerevisiae* and do not localize to LDs. Early biochemical studies of Atf1 and −2 suggest that enzyme activity is membrane dependent. Purification in the presence of non-ionic detergents (e.g., hepthyl thioglucoside, octyl thioglucoside, and Triton-X100) resulted in measurable enzyme activity, while purification in the absence of such detergents resulted in inactive samples [6,20-22]. Due in part to this apparent membrane dependency as well as the hydrophobic nature of the AATase family, the standard activity assay has evolved to include Triton-X100 above the critical micelle concentration [23].

The apparent membrane dependency of Atf1 and −2 activity is interesting in the context of heterologous expression in *E. coli* or other microbial hosts for ester biosynthesis. Reported activities of homologously expressed Atf1 and −2 are moderate, ranging from 0.01 to 10 nmol min^−1^ per mg of protein of whole cell lysate [18,21,22,24], while the activity of orthologs from *Pichia anomla* and *Klyuveromyces lactis* are low (<1 nmol min^−1^ per mg of protein) [25]. Reported activities of strawberry AATases range from 8 – 75 nmol min^−1^ mg of enzyme [26,27]. The activities of Atf1 and −2 were measured in whole cell lysates that contain native LDs to which the enzymes can associate or in the presence of suitable membrane substitutes during purification. The successful metabolic engineering of *E. coli* to produce esters via an AATase pathway indicates that Atf1 and −2 maintain some activity in heterologous environments. In the absence of LDs Atf1 and −2 may associate with the plasma membrane, but the intracellular localization of Atf1 and −2 and other AATases heterologously expressed in *E. coli* and the effects of this localization on activity are not known.

In this work, a series of six AATases from *Saccharomyces* and non-*Saccharomyces* yeasts as well as tomato fruit (*S. lycopersicum*) were overexpressed in *S. cerevisiae* and *E. coli* and compared in terms of their intracellular localization, enzymatic activity, and expression level. The studies revealed that some AATases localize to LDs in *S. cerevisiae* and all studied AATases form enzymatically active aggregates in *E. coli*. Aggregate formation resulted in significantly reduced activities in comparison to activities measured in *S. cerevisiae*. The most active AATases, Atf1 from *S. cerevisiae* and Atf from *S. lycopersicum*, were used to demonstrate an expression strategy to partially recover the lost activity; reduced expression resulted in smaller aggregate size and higher specific activity.

## Results

The strains, plasmids, and AATase genes used in this work are listed in Table 1. The genes include encoded AATases from *Saccharomyces* yeasts (Atf1-S.c, Atf2-S.c, and Atf1-S.p)*,* and the non-*Saccharomyces* yeasts *P. anomala* (Atf-P.a)*,* and *K. lactis* (Atf-K.l)*,* as well as tomato fruit, *S. lycopersicum* (Atf-S.l). Preliminary activity screening from *S. cerevisiae* whole cell lysates with overexpressed AATases revealed that Atf1-S.c has the highest activity towards C2 to C5 alcohols with acetyl-CoA (Additional file 1: Table S1). As such, initial experiments focused on determining the intracellular localization and the enzymatic activity of Atf1-S.c towards ethyl acetate when overexpressed in *S. cerevisiae* and *E. coli*. Importantly, *E. coli* BL-21 without chloramphinicol acetyltransferase (CAT) activity was used, as CAT has been shown to exhibit AATase activity toward ethyl acetate synthesis (Additional file 1: Figure S1 and [16]).

**Table 1:** **Strains, plasmids and primers used in this study**

**Name**	**Description**	**Source**
Strains		
*E. coli* BL21(DE3)	F– ompT gal dcm lon hsdSB(rB- mB-) λ(DE3 [lacI lacUV5-T7 gene 1 ind1 sam7 nin5])	New England Biolabs
*E. coli* BL21(DE3)-RIL plus	F– ompT hsdS(rB– mB–) dcm + Tetr gal λ(DE3) endA Hte [argU ileY leuW Camr]	Agilent Technologies
*S. cerevisiae* BY4742	MATα his3Δ1 leu2Δ0 lys2Δ0 ura3Δ0	GE Healthcare
Plasmids		
pGFP	pET-28b(+) derivative with gfp insertion	This study
pATF1G	pET-28b(+) derivative with atf1(S.c)-gfp insertion	This study
pATF2G	pET-28b(+) derivative with atf2(S.c)-gfp insertion	This study
pSPG	pET-28b(+) derivative with atf(S.p)-gfp insertion	This study
pKLG	pET-28b(+) derivative with atf(K.l)-gfp insertion	This study
pPAG	pET-28b(+) derivative with atf(P.a)-gfp insertion	This study
pSLG	pET-28b(+) derivative with atf(S.l)-gfp insertion	This study
pYPGK	pRS426 derivative; PGK1p-PGK1t	[19]
pYATF1G	pYPGK derivative with atf1(S.c)-gfp insertion	[19]
pYATF2G	pYPGK derivative with atf2(S.c)-gfp insertion	[19]
pYSPG	pYPGK derivative with atf(S.p)-gfp insertion	[19]
pYKLG	pYPGK derivative with atf(K.l)-gfp insertion	[19]
pYPAG	pYPGK derivative with atf(P.a)-gfp insertion	[19]
pYSLG	pYPGK derivative with atf(S.l)-gfp insertion	[19]
Primers		
*gfp*	5'GCTCTAGAAATAATTTTGTTTAACTTTAAGAAGGAGATATACCATGGCTAGCATGACTGGTG3'	This study
	5'CCGCTCGAGTTATTTGTATAGTTCATCCATGCCATG3'	This study
*atf1-S.c-gfp*	5'GCTCTAGAAATAATTTTGTTTAACTTTAAGAAGGAGATATACCATGAATGAAATCGATGAGAAAAATCAG3'	This study
	5'CCGCTCGAGTTATTTGTATAGTTCATCCATGCCATG3'	This study
*atf2-S.c-gfp*	5'GCTCTAGAAATAATTTTGTTTAACTTTAAGAAGGAGATATACCATGGAAGATATAGAAGGATACGAACCACATATCACTC3'	This study
	5'ACGCGTCGACTTATTTGTATAGTTCATCCATGCCATG3'	This study
*atf-S.p-gfp*	5'GCTCTAGAAATAATTTTGTTTAACTTTAAGAAGGAGATATACCATGGAAACAGAAGAAAGCCAATTTAGCAGTATAAC3'	This study
	5'CCGCTCGAGTTATTTGTATAGTTCATCCATGCCATG3'	This study
*atf-K.l-gfp*	5'GCTCTAGAAATAATTTTGTTTAACTTTAAGAAGGAGATATACCATGGGTTCGGTGTGTTTATCATCAAAAAAGTTAG3'	This study
	5'CCGCTCGAGTTATTTGTATAGTTCATCCATGCCATG3'	This study
*atf-P.a-gfp*	5'GCTCTAGAAATAATTTTGTTTAACTTTAAGAAGGAGATATACCATGGTTGTTAAATTCAAAAGCAAAATCAATAACAAAGG3'	This study
	5'CCGCTCGAGTTATTTGTATAGTTCATCCATGCCATG3'	This study
*atf-S.l-gfp*	5'GCTCTAGAAATAATTTTGTTTAACTTTAAGAAGGAGATATACCATGGCTAATATTTTGCCAATTTC3'	This study
	5'CCGCTCGAGTTATTTGTATAGTTCATCCATGCCATG3'	This study

**Figure 2:**
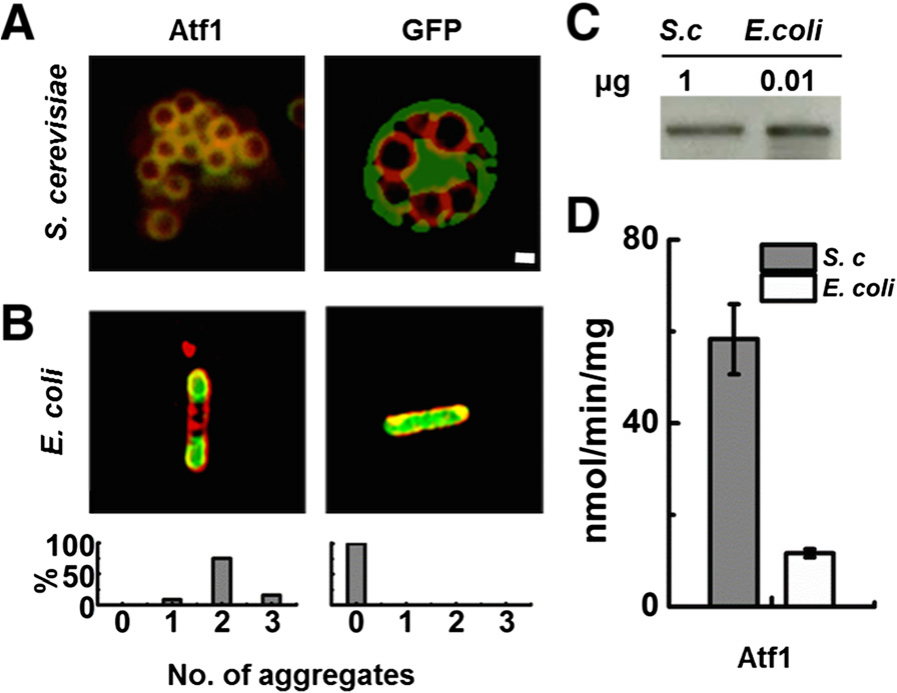
**Expression, intracellular localization, and activity of Atf1 from** ***S. cerevisiae*** **(Atf1-S.c). A)** Fluorescence microscopy images of *S. cerevisiae* co-expressing Atf1-GFP, GFP, and the LD marker Erg6-DsRed. Fluorescence from Erg6-DsRed is shown in red and fluorescence from Atf1-GFP is shown in green. Overlapping Erg6-DsRed and Atf1-GFP signals, indicating co-localization, are shown in yellow. GFP controls show cytosolic localization. Scale bar (1 μm) applies to **A)** and **B)**. **B)** Fluorescent microscopy images of *E. coli* expressing Atf1-GFP and GFP. CellMask™ Orange plasma membrane staining is shown in red and fluorescence from Atf1-GFP and GFP is shown in green. Graphs below the fluorescence images indicate the number of aggregates observed in *E. coli* cells. A minimum of 100 cells were counted from three independent experiments. **C)** Western blot analysis of Atf1-GFP expression in *S. cerevisiae* (S.c) and *E. coli*. **D)** *In vitro* ethyl acetate production from whole cell lysates of Atf1-GFP expressed in *S. cerevisiae* and *E. coli*. Error bars represent standard deviation (n = 3).

In *S. cerevisiae* Atf1-S.c is known to localize to ER in early exponential phase and sort to LDs as cells progress in to stationary phase [19]. Atf1-S.c with a C-terminally fused GFP reporter co-localized with a fluorescently tagged LD marker, Erg6-DsRed, indicating LD localization in *S. cerevisiae* (Figure 2A, left). An overexpressed GFP control localized to the cytosol and fluorescent imaging did not indicate LD localization (Figure 2A, right). When overexpressed in *E. coli*, fluorescence microscopy revealed that GFP is cytosolically expressed and did not form visible aggregates or puncate structures (Figure 2B, right). In contrast, Atf1-S.c. formed aggregates when overexpressed in *E. coli* (Figure 2B, left). Nine percent of observed *E. coli* cells expressing Atf1-S.c. had one aggregate, 75% had two aggregates, and 16% had three aggregates, while no cells were observed with zero aggregates or four or more aggregates (Figure 2B, bottom). No *E. coli* cells expressing Atf1-S.c were observed to have an expression pattern similar to the GFP control, which showed fluorescence throughout the cell. Western blot analysis of protein expression in *S. cerevisiae* and *E. coli* showed that Atf1-S.c with a C-terminally fused GFP reporter expressed upwards of 100-fold more in *E. coli* than in *S. cerevisiae* (Figure 2C). Whole cell lysate activity assays of Atf1-S.c expressed in *S. cerevisiae* reached 58 ± 8 nmol min^−1^ mg^−1^ of protein. Despite the difference in expression levels, *E. coli* whole cell lysate activity assays where limited to 11.6 ± 0.1 nmol min^−1^ mg^−1^ of protein (Figure 2D).

**Figure 3:**
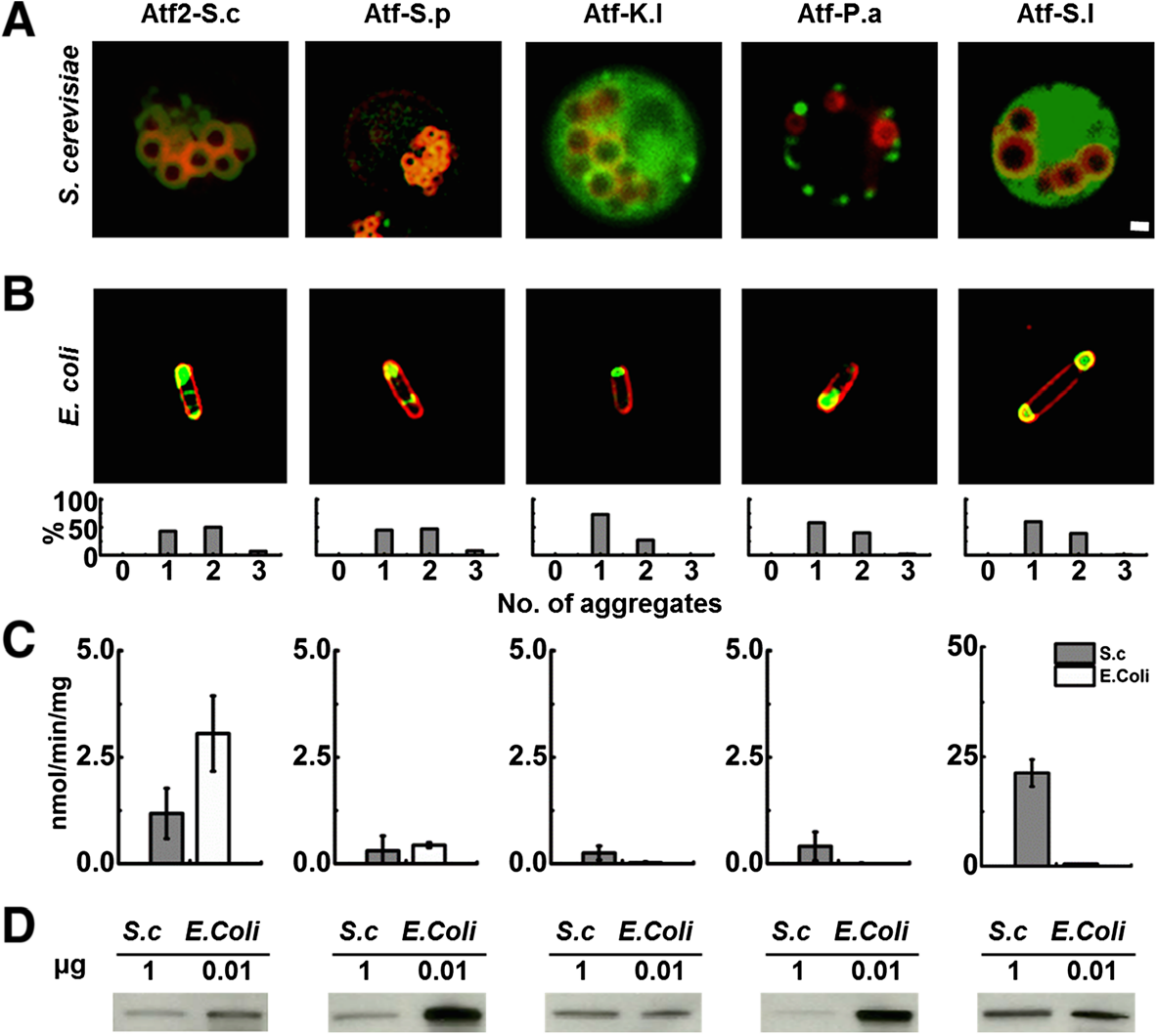
**Expression, intracellular localization, and activity of AATases. A)** Fluorescence microscopy images of *S. cerevisiae* co-expressing AATase-GFP and the LD marker Erg6-DsRed. Expressed AATases include Atf2 from *S. cerevisiae* (Atf2-S.c), and AATases from *S. pastorianus* (Atf-S.p), *K. lactis* (Atf-K.l), *P. anomala* (Atf-P.a), and *S. lycopersicum* (Atf-S.l). Fluorescence from Erg6-DsRed is shown in red and fluorescence from AATase-GFP is shown in green. Overlapping Erg6-DsRed and GFP signals, indicating co-localization, are shown in yellow. Scale bar (1 μm) applies to **A)** and **B)**. **B)** Fluorescent microscopy images of *E. coli* expressing AATases. CellMask™ Orange plasma membrane staining is shown in red and fluorescence from AATase-GFP is shown in green. Graphs below the fluorescence images indicate the number of aggregates observed in *E. coli* cells. A minimum of 100 cells were counted from three independent experiments. **C)** *In vitro* ethyl acetate production from whole cell lysates of AATase-GFP expressed in *S. cerevisiae* (S.c) and *E. coli*. Error bars represent standard deviation (n = 3). **D)** Western blot analysis of AATase-GFP expression in *S. cerevisiae* and *E. coli*.

Similar intracellular localization results were observed with other studied AATases (Figure 3A,B). Atf2-S.c and Atf-S.p localized to LD in *S. cerevisiae* and formed aggregates when overexpressed in *E. coli*. Fluorescent microscopy imaging revealed that Atf-K.l with C-terminally fused GFP was soluble in *S. cerevisiae,* but formed aggregates in *E. coli*. Punctate structures were observed with Atf-P.a in *S. cerevisiae* and, similar to the other yeast AATases, formed aggregates in *E. coli*. Finally, Atf-S.l from tomato fruit appeared to localize homogenously throughout the cytosol of *S. cerevisiae*, but formed aggregates in *E. coli*. In each case, the majority of *E. coli* cells contained one or two aggregates of a given AATase with less than 8% of cells containing three aggregates (Figure 3B, bottom). All observed *E. coli* cells contained at least one aggregate and no cells were observed to contain more than three aggregates.

Whole cell lysate assays revealed that all AATases exhibited measurable activity when expressed in *S. cerevisiae* and *E. coli* (Figure 3C). Atf2-S.c activity in *S. cerevisiae* lysate was 1.2 ± 0.6 nmol min^−1^ mg^−1^, lower than the 3.1 ± 0.9 nmol min^−1^ mg^−1^ observed in *E. coli* lysate. Atf-S.p had activities of 0.3 ± 0.3 and 0.4 ± 0.1 nmol min^−1^ mg^−1^ in *S. cerevisiae* and *E. coli*, respectively. The activities of Atf-K.l and Atf-P.a reached 0.3 ± 0.2 and 0.4 ± 0.3 nmol min^−1^ mg^−1^ in *S.cerevisiae*, but only reached 0.02 ± 0.02 and 0.001 ± 0.001 nmol min^−1^ mg^−1^ in *E. coli*, respectively. Atf-S.l activity in *E. coli* lysate was limited at 0.5 ± 0.2 nmol min^−1^ mg^−1^; however, in *S. cerevisiae* lysate Atf-S.l exhibited activity of 21 ± 3 nmol min^−1^ mg^−1^, second only to the activity of Atf1-S.c. Western blot analysis revealed that in all cases AATase expression in *E. coli* was at least 100-fold greater than expression in *S. cerevisiae* (Figure 3D). In this context, normalization of activity to *S. cerevisiae* expression levels revealed that AATase activity in *E. coli* whole cell lysates for all AATases studied here is less than or equal to 0.08 nmol min^−1^ mg^−1^ of protein.

**Figure 4:**
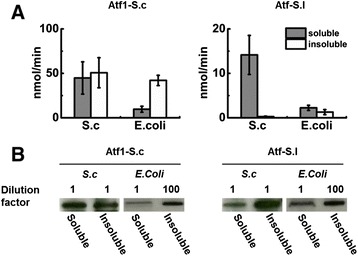
**Atf1-S.c and Atf-S.l activity in soluble and insoluble cell fractions. A)** Comparison of AATase activity in soluble and insoluble fractions from *S. cerevisiae* (S.c) and *E. coli*, Atf1-S.c (left) and Atf-S.l (right). Error bars represent standard deviation (n = 3). **B)** Western blot analysis of soluble and insoluble fractions of Atf1-S.c and Atf-S.l from *S. cerevisiae* and *E. coli* expression.

The ethyl acetate activities of Atf1-S.c and Atf-S.l when expressed in *S. cerevisiae* were significantly greater than the activities of all other studied orthologs. As such, subsequent experiments focused on these two enzymes. To determine if the AATase aggregates formed in *E. coli* were insoluble and active, we measured the activity of soluble and insoluble protein fractions after fractionation by centrifugation. Figure 4A shows that soluble lysate fractions maintained high activity for both Atf1-S.c and Atf-S.l in both hosts (Aft1-S.c: 45 ± 18 and 10 ± 3 nmol min^−1^ in *S. cerevisiae* and *E. coli*, respectively; Atf-S.l: 14 ± 4 and 2.2 ± 0.6 nmol min^−1^ in *S. cerevisiae* and *E. coli*, respectively). Atf1-S.c showed higher activity in the insoluble fractions of both *S. cerevisiae* and *E. coli* (50 ± 17 and 42 ± 6 nmol min^−1^, respectively). In contrast, the insoluble fractions containing overexpressed Atf-S.l showed measurable, but minimal activity (0.25 ± 0.12 and 1.3 ± 0.6 nmol min^−1^ mg^−1^ in *S. cerevisiae* and *E. coli*, respectively). The comparison of activity from *S. cerevisiae* and *E. coli* lysates is not complete without a similar comparison of expression levels in each host (Figure 4B). Western blots of *S. cerevisiae* lysates showed that Atf1-S.c separated equally between the soluble and insoluble fractions, but Atf-S.l separated strongly to the insoluble fraction. When expressed in *E. coli*, both Atf1-S.c and Atf-S.l were largely insoluble. A 100-fold dilution of the insoluble fraction of *E. coli* overexpressing Atf1-S.c and Atf-S.l resulted in western blot bands of near equal intensity to the soluble fractions with no dilution.

**Figure 5:**
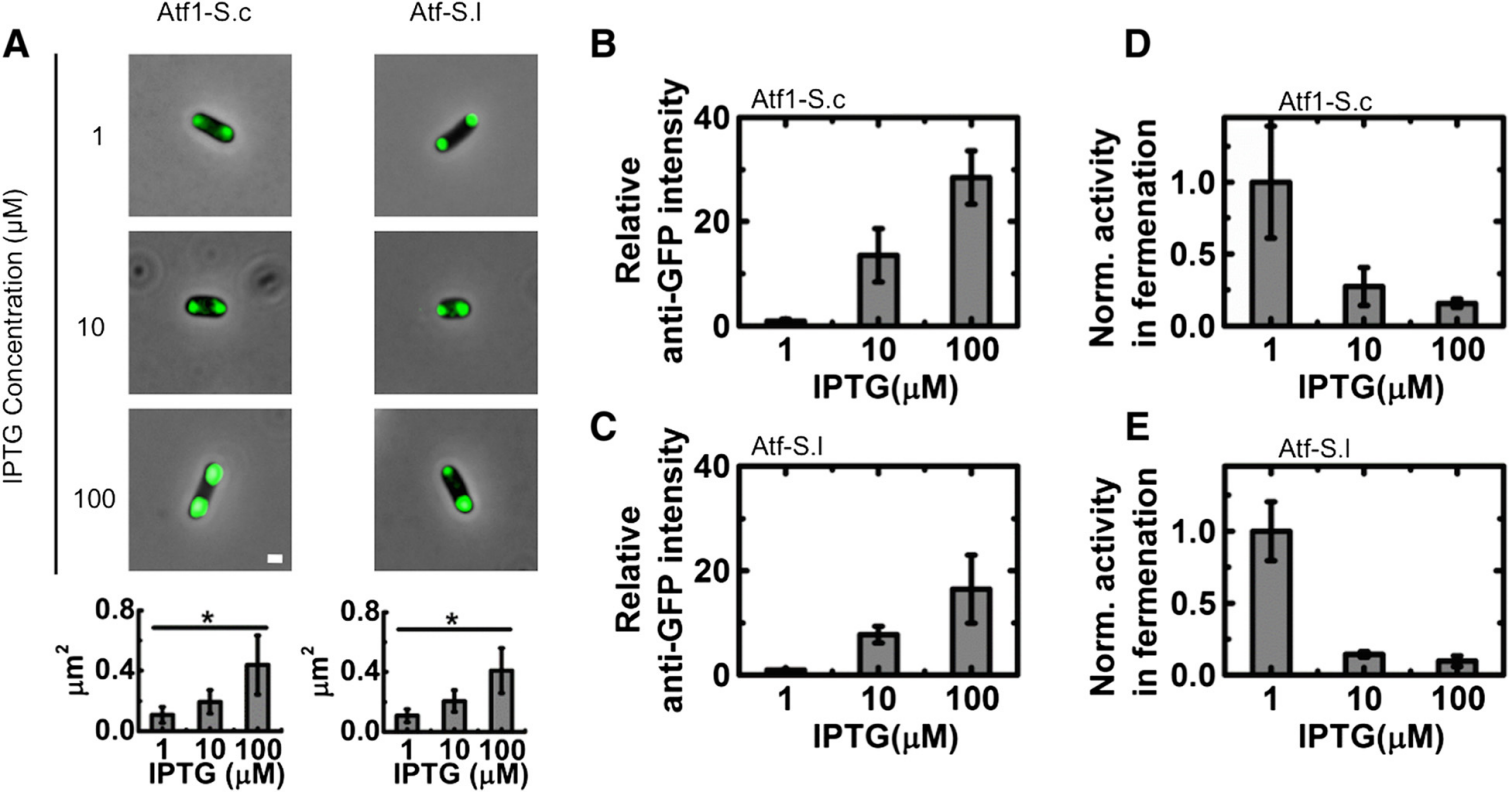
**Reduced AATase expression decreases aggregates size in** ***E. coli*** **fermentations. A)** Fluorescence microscopy images and aggregate size measurement of Atf1-S.c and Atf-S.l in *E. coli*. Scale bar is 1 μm. Error bars represent standard deviation (n = 100), * p < 0.05. **B, C)** Relative expression levels of Atf1-S.c **(B)** and Atf-S.l **(C)** in *E. coli* as judged by western blot analysis. Expression levels are normalized to the intensity of anti-GFP signal from *E. coli* induced with 1 μM IPTG. **D, E)** Normalized AATase activity from Atf1-S.c **(D)** and Atf-S.l **(E)** during 24 hour *E. coli* fermentations at 30°C. Activities are normalized to the lowest expression level, IPTG induction of 1 μM. Error bars represent standard deviation (n = 3).

To further investigate the relationship between AATase overexpression in *E. coli* and enzymatic activity we modulated expression levels using a standard inducible T7 promoter system. Analysis of fluorescence microscopy images of *E. coli* expressing Atf1-S.c with C-terminal GFP tag revealed that intracellular aggregate size increased from 0.11 ± 0.05 μm^2^ when induced with 1 μM IPTG to 0.19 ± 0.08 μm^2^ with 10 μM IPTG (Figure 5A and Additional file 1: Figure S2). Aggregate size further increased to 0.44 ± 0.20 μm^2^ with 100 μM IPTG. Coincident with increased induction and aggregate size were Atf1-S.c. expression levels (Figure 5B and Additional file 1: Figure S3). Induction with 10 and 100 μM IPTG resulted in 13.5 ± 5.1 and 28.5 ± 5.1 fold increases in protein expression over levels observed with 1 μM IPTG induction. Similar aggregate size and protein expression levels were observed with Atf-S.l. Under low induction conditions, aggregates of Atf-S.l with a C-terminal GFP tag were found to be 0.11 ± 0.04 μm^2^. Aggregate size increased to 0.20 ± 0.07 and 0.41 ± 0.15 μm^2^ when induced with 10 and 100 μM IPTG, respectively. Quantification of western blots showed that protein expression increased by 7.8 ± 1.6 and 16.5 ± 6.6 fold with increased induction levels of 10 and 100 μM IPTG, respectively (Figure 5C).

The result of reduced expression level and aggregate size was an increase in specific AATase activity during fermentation. Figures 5D shows Atf1-S.c activity in terms of ethyl acetate produced during a 24-hour fermentation normalized to the activity at the lowest protein expression level (i.e., induction with 1 μM IPTG). Increased expression of Atf1-S.c reduced specific activity by 72.6 ± 13.1% and 84.2 ± 2.7% with 10 and 100 μM IPTG, respectively (Figure 5D). Despite the considerable decrease in specific activity, high expression levels of Atf1-S.c resulted in increased ethyl acetate production under fermentation. For Atf1-S.c, the amount of ethyl acetate produced in 24-hours of fermentation increased from 1.35 ± 0.10 to 4.78 ± 0.28 and 6.74 ± 0.24 mg/L with induction from 1 to 10 and 100 μM IPTG, respectively, while the amounts of ethanol produced under the same conditions were 0.87 ± 0.07, 0.89 ± 0.04, and 0.94 ± 0.01 g/L, respectively (Additional file 1: Figure S4). It is important to note that culture density measured by absorbance at 600 nm, A_600_, was unchanged with varied induction levels when expressing Atf1-S.c and a small but statistically significant reduction in A_600_ was observed between the lowest and highest induction levels when expressing Atf-S.l (A_600_ of 3.64 ± 0.44 and 2.48 ± 0.36, respectively; Additional file 1: Figure S5). As shown in Figure 5B, Atf1-S.c expression increased by upwards of 28-fold under the same conditions, considerably more than the fold increase in ethyl acetate production, indicating that increasing enzyme expression did not result in a proportional increase in ester synthesis. Importantly, the increase in ethyl acetate production with increased expression of Atf1-S.c suggested that AATase activity is rate limiting for the conversion of ethanol to ethyl acetate and that the observed loss in activity is due to the enzyme and not an upstream pathway bottleneck. The low conversions of ethanol to ethyl acetate are also supportive of Atf1-S.c activity as rate limiting.

**Figure 6:**
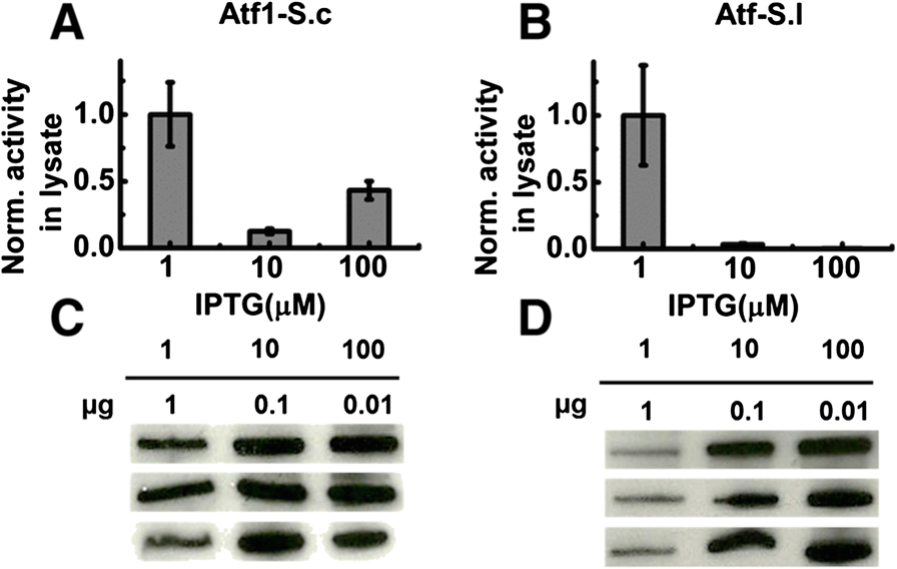
**Reduced AATase expression increases specific activity in whole cell lysate assays. A,B)** Atf1-S.c **(A)** and Atf-S.l **(B)** activity in *E. coli* whole cell lysates normalized to the expression level from induction with 1 μM IPTG. Error bars represent standard deviation (n = 3). **C,D)** Western blot analysis of Atf1-S.c **(C)** and Atf-S.l **(D)** expression levels in *E. coli* with inductions of 1, 10, and 100 μM IPTG.

During 24 hours of *E. coli* fermentation with low expression of Atf-S.l, 0.30 ± 0.09 mg/L of ethyl acetate and 0.95 ± 0.20 g/L of ethanol were produced; there was no statistically significant different in the production of either compound with increased induction (Additional file 1: Figure S4). The low levels of ethyl acetate production in comparison to fermentation with Atf1-S.c expressing *E. coli* were expected due to lower activity of Atf-S.c ; however, varying expression levels of Atf-S.l produced a similar trend in normalized specific activity to that observed with Atf1-S.c. At higher expression levels specific activity was reduced by 85.6 ± 2% and 90.3 ± 4% with inductions of 10 and 100 μM IPTG, respectively (Figure 5E). To confirm the effects of varying expression on activity, whole cell lysate assays and corresponding western blots were performed. For both Atf1-S.c and Atf-S.l the relative protein levels in whole cell lysates increased with increased induction (Figure 6A,B). Similar to the fermentation studies, the increased protein levels did not result in a proportional increase in ethyl acetate synthesis and normalized specific activity decreased significantly with induction levels of 10 and 100 μM IPTG. For Atf1-S.c the highest protein levels resulted in less of a reduction in activity then with intermediate protein levels (residual activities of 12.4 ± 2.0% and 43.3 ± 6.9% for 10 and 100 μM IPTG, respectively). For Atf-S.l increased protein levels due to induction with 10 and 100 μM IPTG measured specific activities were less than 5% of the specific activity from 1 μM IPTG induction.

## Discussion

A major challenge in engineering metabolic pathways, including ester biosynthesis, is pathway optimization including the alleviation of kinetics bottlenecks and balancing the kinetic capacity of pathway steps. One approach to maximizing flux is pathway engineering to alter the expression levels of each step [28,29]. Enzyme engineering to improve the kinetics of key steps is also critical [30]. The difficulty of these approaches is compounded when native intracellular localization, activity, and expression of key enzymes are effected by heterologous expression. Flux analysis and pathway modeling are also negatively affected because experimentally determined kinetic parameters used in modeling do not accurately translate to those exhibited *in vivo*. The AATases studied here are prime examples of these problems as heterologous expression in *E. coli* resulted in losses in specific activity and in some cases a loss of intracellular localization.

Homologous expression of Atf1-S.c in *S. cerevisiae* revealed high activity in whole cell lysates and native localization to LDs. Heterologous expression in *E. coli* was approximately 100-fold higher than in *S. cerevisiae*, but whole cell lysate assays revealed significantly reduced specific activity and *in vivo* fluorescence imaging revealed a loss of membrane localization (Figure 2). These results were consistent with Atf2-S.c from *S. cerevisiae* and Atf-S.p from *S. pastorianus*. Enzymes localized to LD in *S. cerevisiae,* but membrane localization was not observed in *E. coli* (Figure 2A,B). Despite high expression in *E. coli*, specific activities of Atf2-S.c and Atf-S.p in *E. coli* cell lysates were significantly less than observed in *S. cerevisiae* lysates. AATases from *K. lactis*, *S. lycopersicum*, and *P. anomala* did not localized to LD or other membranes when expressed in *S. cerevisiae* and did not localized to membranes in *E. coli*. Again, expression in *E. coli* was high and specific activities measured in whole cell lysates were significantly reduced in comparison with activities measured in *S. cerevisiae* lysates. Previous work investigating the localization of AATases in *S. cerevisiae* has shown that minimal modifications to the C-terminus did not affect the membrane localization of Atf1-S.c, Atf2-S.c, or Atf-S.p and that the C-terminal GRF reporter did not prevent localization [19]. Conserved N- and C-terminal amphipathic helices were identified as necessary for AATase membrane localization, motifs that are lacking in Atf-K.l, −P.a, and -S.l. In this context, it is unlikely that the C-terminal GFP reporter alters membrane localization; however, this remains as a possible explanation for the lack of AATase localization in *S. cerevisiae*.

A contributing factor to the loss of activity was AATase aggregation in *E. coli*. Fluorescence imaging of *E. coli* cells revealed that in all cases expressed AATase formed one or more cytosolic aggregates (Figure 2B, 3B). Importantly, the aggregates were functional. We expected that loss of tertiary structure in inclusion bodies would destroy protein function, but in all cases C-terminally fused GFP maintained fluorescence and whole cell lysates exhibited AATase activity suggesting that AATases expressed in *E. coli* were not misfolded [31]. Moreover, analysis of fractionated *E. coli* lysates containing Atf1-S.c or Atf-S.1 demonstrated measurable activity in both the soluble and insoluble fractions (Figure 4). Common to the AATase family are short stretches of hydrophobic amino acids that are a possible source of protein aggregation [8,18]. Specific to AATases from *S. cerevisiae* and *S. pastorianus,* the N- and C-terminal amphipathic helices that function as ER and LD membrane anchors may also be a source of aggregation [19]. Regardless of mechanism(s), the results of heterologous expression in *E. coli* were clear, the formation of AATase active aggregates. The loss of activity was most likely due to a combination of diffusion limitations and blocked active sites. The results demonstrating increased specific activity at reduced expression levels supports these conclusions (Figures 5 and 6). Reduced expression of Atf1-S.c and Atf-S.l in *E. coli* produced smaller aggregates and increased measures of specific activity both in whole cell lysate assays and under fermentation conditions. Reduction in aggregate size increases the ratio of surface to interior proteins thus minimizing blocked active sites. It is also possible that activity in aggregates was due to the disruption of active protein conformation, an effect that is also likely decreased with aggregate size. An alternative explanation is that AATase solubility was increased at lower induction levels, an effect that has previously been demonstrated with single chain antibodies expressed in an *E. coli* host [32]. An additional means of decreasing expression and increasing solubility is culturing at lower temperatures; however, fluorescence microscopy revealed that expression in *E. coli* at 20 and 30°C produced aggregates (Additional file 1: Figure S6).

Biochemical studies of Atf1 from *S. cerevisiae* (Atf1-S.c) have suggested that activity is membrane-dependent [6,20-22]. The studies presented here confirm that Atf1-S.c, Atf2-S.c and Atf-S.p localize to LDs in *S. cerevisiae*, but do not localize to membranes in *E. coli*. The loss of membrane localization did not eliminate activity towards ethyl acetate suggesting that these AATases are not strictly membrane dependent with respect to activity (Figures 2 and 3). Published purification protocols for Atf1-S.c use surfactants or non-ionic detergents and in their absence activity is significantly reduced. In our hands, nickel-affinity chromatography purification of Atf1-S.c resulted in AATase active samples (Additional file 1: Figure S7), thus supporting the claim that membrane localization is not necessary for enzymatic activity. This lack of strict membrane dependency provides an explanation as to why metabolic engineering of *E. coli* to synthesize short chain volatile esters has been successful, and our analysis of AATase expression and activity in *S. cerevisiae* and *E. coli* hosts provides important information for future metabolic engineering of ester biosynthesis.

## Conclusions

Ester biosynthesis is a promising new target for metabolic engineering. The market price of ethyl and butyl acetates are upwards of $1500 per tonne (http://www.icis.com/), and the total market value for flavor and fragrance compounds is greater than $16 billions (http://www.ialconsultants.com/). The high volatility of shorter chain esters such as ethyl acetate is also attractive from a separation perspective, as high volatility facilitates separation from fermentation broths [33]. The results presented here demonstrate that microbial host selection is critical to ethyl acetate biosynthesis through AATase activity. Heterologous AATase expression in *E. coli* resulted in significantly decreased specific activity in comparison to activity measure in *S. cerevisiae*. All studied AATases formed aggregates when expressed in *E. coli* and any membrane localization in *S. cerevisiae* was lost in *E. coli*. One solution to minimizing the loss of activity is reduced expression levels in *E. coli*, which resulted in smaller aggregate size and increased specific activity in comparison to high overexpression. The effects of host selection on AATase expression and activity described here are interesting in that they provide evidence that the AATases studied here are not strictly membrane dependent with respect to activity and are important when considering metabolic engineering strategies for ester biosynthesis.

## Methods

### Strains, plasmids, and culture conditions

Strains and plasmids used in this work are shown in Table 1. *E. coli* strains were grown in LB medium containing 30 μg/mL kanamycin. *S.cerevisiae* strains were prepared as previously described [19], and were grown in synthetic minimal (SD) medium containing 0.67% yeast nitrogen base (Becton-Dickinson), amino acid supplements (Sunrise), and 2% glucose. Expression in *E. coli* was induced by adding IPTG at OD_600_ of 0.4.

### Preparation of whole cell lysate

Cells were harvested by centrifugation at 3,500 rpm for 5 min at 4°C and washed twice with 100 mM potassium phosphate buffer (pH 7.4) containing 2 mM magnesium chloride. Equal volumes of wet cell pellets and 425–600 μm acid-washed glass beads (Sigma-Aldrich, G8772) were added to a 15 mL tube and resuspended in 1 mL ice-cold lysis buffer (100 mM potassium phosphate buffer, 2 mM magnesium chloride, 2 mM DTT, and protease inhibitor). The cells were disrupted by vortexing 10 times for 30 s. After each vortexing the suspension was kept on ice for 30 s. The beads were removed by centrifugation at 500 g for 5 min at 4°C, and the supernatant was decanted to a cold 2 mL tube. The protein concentrations of whole cell lysates were determined by Thermo Scientific Pierce 660 nm Protein Assay.

### Enzyme activity assays

AATase activity was measured using ethanol and acetyl-CoA as substrates. A reaction mixture contains 100 mM potassium phosphate (pH 7.4), 500 mM ethanol and 0.5 mM acetyl-CoA and 100 μg lysate was used. After incubation at 30°C for 0.5 hours, the reaction was stopped by the addition 60 μmol H_2_SO_4_. 100 μg of 1-pentanol was added as an internal standard and 1 g NaCl was added to reduce the solubility of ethyl acetate. The concentration of ethyl acetate produced was measured by headspace gas chromatography. To determine the activity of soluble and insoluble cell fractions, whole cell lysates were centrifuged at 15,000 rpm for 20 min at 4°C. The supernatant was isolated and taken as the soluble fraction. The pellet was washed twice with lysis buffer before re-suspending in lysis buffer. The activity of the re-suspended pellet and soluble fraction were measured as described above.

### Fermentations

*E. coli* strains were grown in Terrific Broth containing 2% glucose and 30 μg/mL kanamycin with IPTG induction at OD_600_ of 0.4. Cells were cultured anaerobically in 125-mL screwed cap flask at 30°C for 24 hours on a rotary shaker. The shake flask headspace was purged with nitrogen prior to incubation.

### Ethyl acetate detection

Produced ethyl acetate was quantified by a headspace gas chromatography with a flame ionization detector (Agilent Technologies 7890A GC with CTC-PAL headspace mode injector). The separation of volatile compounds was carried out by Rtx®-1 column (30 m, 0.32 mmID, 5 μm film thickness; RESTEK) with helium as carrier gas. GC oven temperature was initially at 75°C for 7 min and increased with a gradient of 30°C/min until 175°C, followed by a gradient of 50°C/min until 275°C. The injector and detector were held at 275°C. 1 mL headspace gas was injected to the GC and 1-pentanol was used as internal standard.

### Fluorescent microscopy and image analysis

Cells were observed as described in [19]. Briefly, an Olympus BX51 microscope (UPlanFL 100X 1.30 oil-immersion objective lense, mercury lamp) with Q-Imaging Retiga Exi CCD camera was used to capture images. CellSens Dimension 1.7 software (Olympus) was used to process images. Image J software was used to measure protein aggregate size. Quantitative values of aggregates size and number are from a minimum of 100 cells. Statistical analysis of aggregate size was accomplished by one-way analysis of variance. A p value <0.05 was applied for statistical significance.

### Western blot analysis and quantification of protein expression

Western blots were performed using standard procedure. Protein extracts were loaded on Any kD™ Mini-PROTEAN® TGX™ Gel (Bio-Rad) and run at 150 V for 1 hour. Samples were electrophoretically transferred to a PVDF membrane at 25 V overnight. Membranes were blocked with 5% non-fat milk in TBST buffer for 1 hour at room temperature and incubated with GFP Rabbit Serum Polyclonal Antibody (Life Technologies) diluted to 1: 20000 in TBST buffer with 1% non-fat milk. Goat Anti-Rabbit IgG-HRP (Life Technologies) diluted to 1: 10000 was added as secondary antibody and incubated at room temperature for 0.5 hours. After washing with TBST, HRP substrate (Bio-Rad) was used for signal detection. Image-J software was used to quantify band intensity.

## Additional file

Additional file 1: Table S1. AATases activity towards different alcohols and acetyl-CoA. Data shown is mean (n=3). Not detected (n.d.). **Figure S1.** Background ethyl acetate synthesis from *E. coli* strains BL21(DE3)-RIP plus and BL21(DE3). The RIL strain expresses chloramphinicol acetyltransferase, which exhibits AATase activity towards ethanol and acetyl-CoA. **Figure S2.** Image J analysis of protein aggregate size in *E. coli*. **Figure S3.** Western blots of Atf1-S.c and Atf-S.l expressed in *E. coli* with increasing IPTG concentration. **Figure S4.** Ethyl acetate (EA) and ethanol (EtOH) production of *E. coli* harboring plasmids pET28-Atf1-S.c and pET28-Atf-S.l induced with different IPTG concentration under fermentation condition. **Figure S5.** A_600_ values of *E. coli* harboring plasmids pET28-Atf1-S.c and pET28-Atf-S.l induced with different IPTG concentration under fermentation condition. **Figure S6.** Fluorescent microscopy imaging of E. coli expressing Atf1-S.c at culture temperatures (temp.) of 20, 30, and 37°C. **Figure S7.** A) Specific activity of purified Atf1-S.c with C-terminal his tag. B) SDS-PAGE gel of purified Atf1-S.c. The red arrow points to Atf1.
