# Supplementary material for: Microbial host selection affects intracellular localization and activity of alcohol-O-acetyltransferase
Source: Microb Cell Fact. 2015 Mar 17;14:35. doi: 10.1186/s12934-015-0221-9 (PMC4367896; doi:10.1186/s12934-015-0221-9)
Supplement: Additional file 1: Table S1. — AATases activity towards different alcohols and acetyl-CoA. Data shown is mean (n=3). Not detected (n.d.). Figure S1. Background ethyl acetate synthesis from E. coli strains BL21(DE3)-RIP plus and BL21(DE3). The RIL strain expresses chloramphinicol acetyltransferase, which exhibits AATase activity towards ethanol and acetyl-CoA. Figure S2. Image J analysis of protein aggregate size in E. coli. Figure S3. Western blots of Atf1-S.c and Atf-S.l expressed in E. coli with increasing IPTG concentration. Figure S4. Ethyl acetate (EA) and ethanol (EtOH) production of E. coli harboring plasmids pET28-Atf1-S.c and pET28-Atf-S.l induced with different IPTG concentration under fermentation condition. Figure S5. A600 values of E. coli harboring plasmids pET28-Atf1-S.c and pET28-Atf-S.l induced with different IPTG concentration under fermentation condition. Figure S6. Fluorescent microscopy imaging of E. coli expressing Atf1-S.c at culture temperatures (temp.) of 20, 30, and 37°C. Figure S7. A) Specific activity of purified Atf1-S.c with C-terminal his tag. B) SDS-PAGE gel of purified Atf1-S.c. The red arrow points to Atf1. [file 12934_2015_221_MOESM1_ESM.docx]

**Supporting Information**

**Microbial host selection affects intracellular localization and activity of alcohol-O-acetyltransferase**

Jie Zhu^1^, Jyun-Liang Lin^2^, Leidy Palomec^2^, and Ian Wheeldon^2*^

1. Department of Biochemistry, University of California, Riverside USA 92521
2. Department of Chemical and Environmental Engineering, University of California, Riverside USA 92521

Table S1

Figure S1

Figure S2

Figure S3

Figure S4

Figure S5

Figure S6

Figure S7

**Table S1.** AATases activity towards different alcohols and acetyl-CoA. Data shown is mean (n=3). Not detected (n.d.).


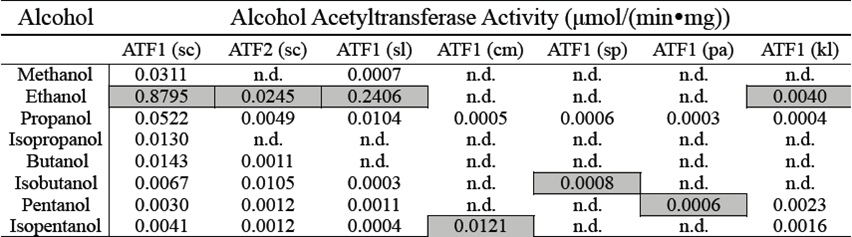


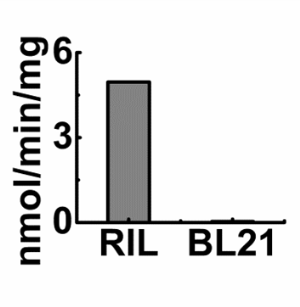


**Figure S1.** Background ethyl acetate synthesis from *E. coli* strains BL21(DE3)-RIP plus and BL21(DE3). The RIL strain expresses chloramphinicol acetyltransferase, which exhibits AATase activity towards ethanol and acetyl-CoA.


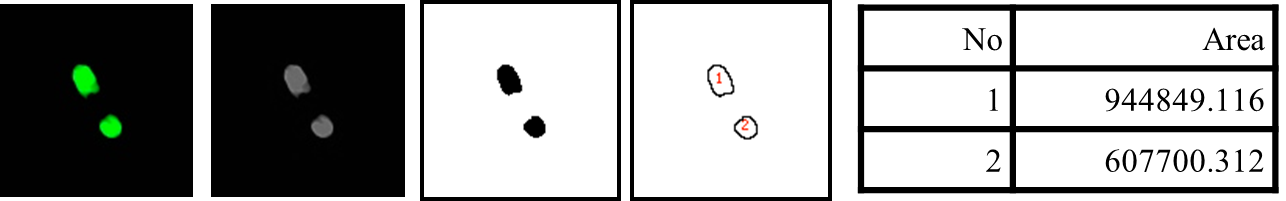


**Figure S2.** Image J analysis of protein aggregate size in *E. coli*.


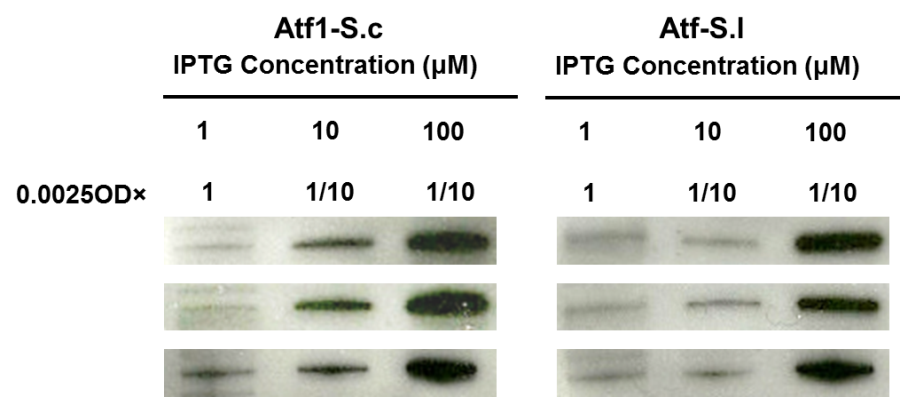


**Figure S3.** Western blots of Atf1-S.c and Atf-S.l expressed in *E. coli* with increasing IPTG concentration.


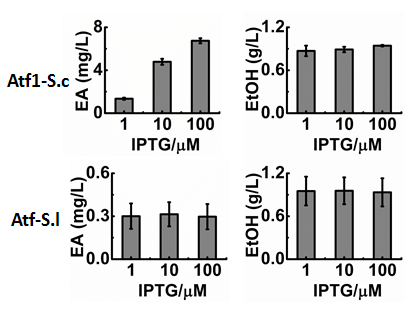


**Figure S4.** Ethyl acetate (EA) and ethanol (EtOH) production of *E. coli* harboring plasmids pET28-Atf1-S.c and pET28-Atf-S.l induced with different IPTG concentration under fermentation condition.


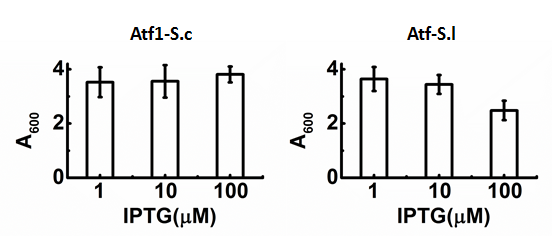


**Figure S5.** A_600_ values of *E. coli* harboring plasmids pET28-Atf1-S.c and pET28-Atf-S.l induced with different IPTG concentration under fermentation condition.


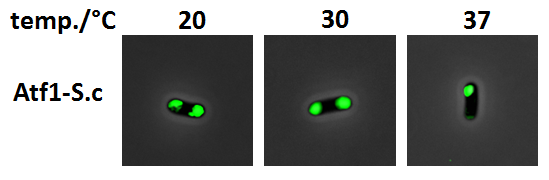


**Figure S6.** Fluorescent microscopy imaging of E. coli expressing Atf1-S.c at culture temperatures (temp.) of 20, 30, and 37 °C.


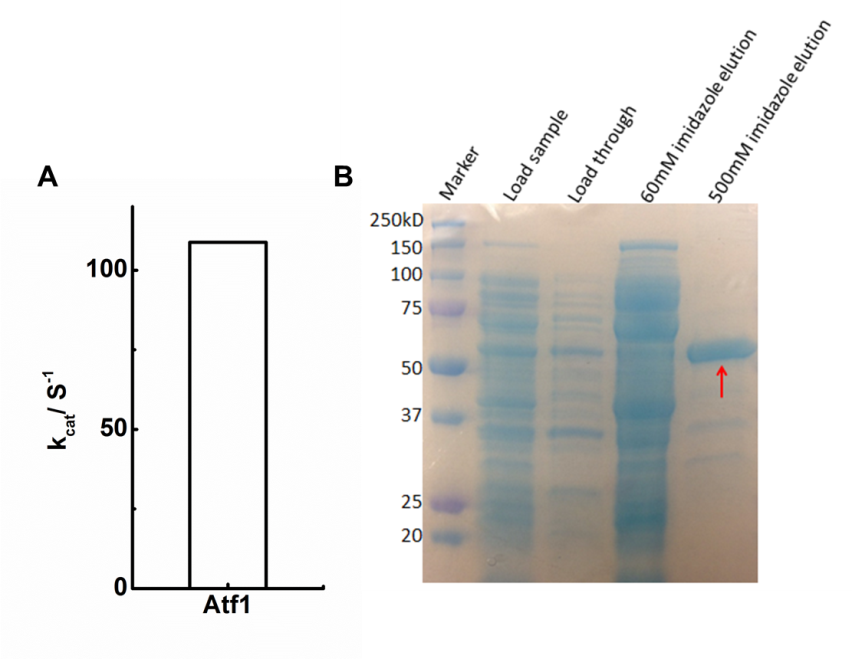


**Figure S7.** A) Specific activity of purified Atf1-S.c with C-terminal his tag. B) SDS-PAGE gel of purified Atf1-S.c. The red arrow points to Atf1.
